# A Liver Index and its Relationship to Indices of HCC Aggressiveness

**DOI:** 10.4172/2329-6771.1000178

**Published:** 2016-09-05

**Authors:** Brian I Carr, Vito Guerra, Edoardo G Giannini, Fabio Farinati, Francesca Ciccarese, Gian Ludovico Rapaccini, Maria Di Marco, Luisa Benvegnù, Marco Zoli, Franco Borzio, Eugenio Caturelli, Alberto Masotto, Franco Trevisani

**Affiliations:** 1 Izmir Biomedicine and Genome Center, Dokuz Eylul University, Turkey and Lusaka Apex Medical University, Zambia; 2 Department of Clinical Trials and Epidemiology, IRCCS de Bellis, Castellana Grotte, Italy; 3 Department of Internal Medicine, Gastroenterology Unit, University of Genoa, Italy; 4 Department of Surgical Science and Gastroenterology, Gastroenterology Unit, University of Padua, Italy; 5 Division of Surgery, Policlinico San Marco, Zingonia, Italy; 6 Internal Medicine and Gastroenterology Unit, Catholic University of Rome, Italy; 7 Division of Medicine, Azienda Ospedaliera Bolognini, Seriate, Italy; 8 Department of Clinical and Experimental Medicine, Medical Unit, University of Padua, Italy; 9 Department of Medical and Surgical Science, Internal Medicine Unit, Alma Mater Studiorum, University of Bologna, Italy; 10 Department of Medicine, Internal Medicine and Hepatology Unit, Ospedale Fatebenefratelli, Milan, Italy; 11 Gastroenterology Unit, Ospedale Belcolle, Viterbo, Italy; 12 Gastroenterology Unit, Ospedale Sacro Cuore Don Calabria, Negrar, Italy; 13 Department of Medical Surgical Sciences, Medical Semiotics Unit, Alma Mater Studiorum, University of Bologna, Italy

**Keywords:** HCC, Aggressiveness index, Survival, Liver function

## Abstract

A Hepatocellular (HCC) Aggressiveness Index was recently constructed, consisting of the sum of the scores for the 4 clinical parameters of maximum tumor size, multifocality, presence of portal vein thrombus and blood alphafetoprotein levels. It was observed that there was an association with several liver function tests. We have now formed a Liver Index from the 4 liver parameters with the highest hazard ratios with respect to HCC aggressiveness, namely: blood total bilirubin, gamma glutamyl transpeptidase (GGTP), albumin and platelet levels (cirrhosis surrogate). We found that the scores for the Liver Index related significantly to survival, but also to the Aggressiveness Index and to its individual HCC components as well as showing significant trends with the components. These results support the hypothesis that liver function is not only an important prognostic factor in HCC patients, but may also be involved in HCC biology and aggressiveness. Blood albumin, GGTP, albumin and platelet levels were used to create a Liver Index that related significantly to parameters of HCC aggressiveness.

## Introduction

Human hepatocellular carcinoma (HCC) has 4 clinical characteristics that are used to evaluate treatment options and prognosis (in addition to metastasis). They are: maximum tumor diameter (MTD), presence or absence of of tumor multifocality, presence or absence of portal vein thrombus (PVT) and blood alpha-fetoprotein (AFP) level. These 4 parameters were recently integrated into an Aggressiveness Index, with points allotted to the level of each separate parameter and the total were then summed [1]. This approach was then validated in another, much larger dataset [2]. It was also observed that with each score level in the Aggressiveness Index, there was a significant difference in patient survival. Furthermore, there were increased mean values for several common liver function parameters, with increase in the Aggressiveness Index score.

The analysis of a possible relationship between indices of HCC aggressiveness and liver function parameters, has been extended in the current work, by examining whether liver function parameter values in patients with HCC might be useful in predicting HCC aggressiveness. We found that a composite Liver Score, comprising blood total bilirubin, GGTP, albumin and blood platelet levels (surrogate for cirrhosis) relate to both trends in the individual HCC aggressiveness components, and also to the Aggressiveness Index.

## Methods

### Construction of the liver index

The Liver Index is the sum of the scores for blood GGTP+total bilirubin+albumin+platelet levels. The scores were assigned as follows:

GGTP IU/ml (cut-off): GGTP<100; 100 ≤ GGTP ≤ 200; GGTP>200 levels, were assigned a score of 1, 2, 3 respectively.
Bilirubin mg/dl (cut-off): Bilirubin<1.5; 1.5 ≤ Bilirubin ≤ 2.5; Bilirubin>2.5 levels, were assigned a score of 1, 2, 3 respectively.
Albumin g/dl (cut-off): Albumin>3.5; 2.5 ≤ Albumin ≤ 3.5; Albumin<2.5 levels, were assigned a score of 1, 2, 3 respectively.
Platelets×109/l (cut-off): Platelets<100; 100 ≤ Platelets ≤ 150; Platelets>150 levels, were assigned a score of 1, 2, 3 respectively.

The point scores were derived from dividing the full range of each parameter into terciles, each increasing tercile then being given an increased point of 1, 2 or 3.

The Liver Index score was divided into three groups for Kaplan-Meier Survival and Cox analysis: a, score=4; b, 4<score ≤ 8; and c, score>8.

## Patients and Data Collection

### Data collection

We retrospectively analyzed prospectively-collected data in the Italian Liver Cancer (ITA.LI.CA) study group database of 2706 HCC patients accrued till 2008 at 11 centers [1,2] who had full baseline tumor parameter data, including CT scan information on maximum tumor diameter (MTD), number of tumor nodules and presence of PVT and plasma AFP levels; blood counts; routine blood liver function tests, (total bilirubin, GGTP, albumin); demographics and survival information. ITA.LI.CA database management conforms to Italian legislation on privacy and this study conforms to the ethical guidelines of the Declaration of Helsinki. Approval for this retrospective study on de-identified HCC patients was obtained by the Institutional Review Board of participating centers.

Aggressiveness Index was calculated as the sum of scores:

MTD (cm, in tertiles): MTD<4.5; 4.5 ≤ MTD ≤ 9.6; MTD>9.6; scores 1, 2, 3 respectively;
AFP ng/ml (cut-off): AFP<100; 100 ≤ AFP ≤ 1000; AFP>1000; scores 1, 2, 3 respectively;
PVT (No/Yes): PVT(No); PVT(Yes); scores 1, 3 respectively;
Number of Tumor Nodules: Nodules ≤ 3; Nodules>3; scores 1, 3 respectively.

### Statistical analysis

Mean and SD for continuous variables were used as indices of centrality and dispersion of the distribution.

It was necessary, for non-normally distributed values, for the continuous variables, to use non-parametric methods. We used the Kruskal-Wallis rank test for differences of the parameters among the three categories of the Liver Index, and the Wilcoxon rank-sum (Mann-Whitney) test for the comparisons of the Aggressiveness Index between two categories at a time of the Liver Index score.

The test for trend was used to evaluate the trend of the Aggressiveness Index means among Liver index score categories.

Linear regression and multiple linear regression models were used for the association of the Aggressiveness Index score on the Liver Index score (A), and on each serum variable, Gamma Glutamyl Transpeptidase (GGTP), Total Bilirubin (Bilirubin), Albumin (Alb), and Platelets (Plt), included together in the model (B). The results were presented as coefficients (β) with 95% C.I.

Patient survival between the three categories of the Liver Index score was estimated with the Kaplan-Meier method and comparison of survival was made with the Breslow (generalized Wilcoxon) test. The Breslow test was used, as opposed to the log rank test, due to the large proportion of patients who died early.

The Cox proportional hazards model was applied to evaluate the predictive factors as categories of Liver Index score associated with overall survival. The results were presented as Hazard Ratio (HR) with 95% C.I.

In all models, for Cox regression model, the HR, and for the Linear regression and Logistic regression, the β, represent the variation of the dependent variable, for one-unit variation of the predictor variable considered both as dummy or as continuous variable.

When testing the hypothesis of significant association, p-value was <0.05, two tailed for all analyses. Statistical analysis was performed with StataCorp. 2007. Stata Statistical Software: release 10. College Station, TX: StataCorp LP.

## Results

### Liver index and its relationship to patient survival

The construction of the Liver Index is shown in Table 1, and comprises scores of 1, 2 or 3 which are assigned to the values in an HCC patient for blood levels of GGTP, total bilirubin, albumin and platelet levels.

**Figure 1:**
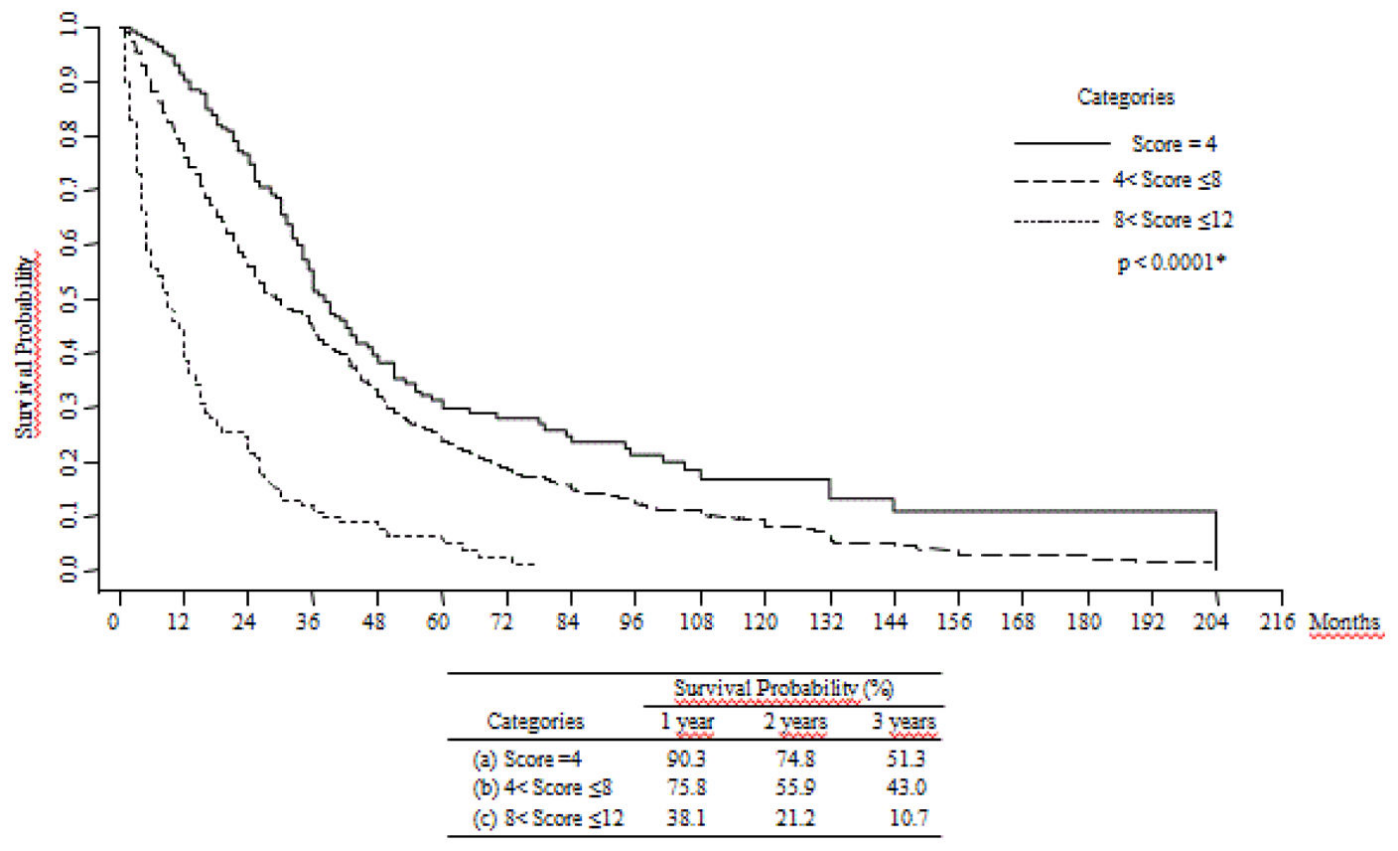
Kaplan-Meier Survival plots between groups of the Liver Index in total cohort. *Wilcoxon (Breslow) test. The Liver Index is the sum of the scores for GGTP+Bilirubin+Albumin+Platelets: GGTP (cut-off): GGTP<100; 100 ≤ GGTP ≤ 200; GGTP>200; for scores 1, 2, 3 respectively. Bilirubin (cut-off): Bil<1.5; 1.5 ≤ Bil ≤ 2.5; Bil>2.5; for scores 1, 2, 3 respectively. Albumin (cut-off): Alb>3.5; 2.5 ≤ Alb ≤ 3.5; Alb<2.5; for scores 1, 2, 3 respectively. Platelet (cut-off): Plt<100; 100 ≤ Plt ≤ 150; Plt>150; for scores 1, 2, 3 respectively.

These 4 parameters were chosen since they had the highest Odds Ratios (OR) in a multiple regression analysis for the HCC aggressiveness Index [2], and they were divided into three categories, each category being given a point score of 1, 2 or 3, respectively. The total Liver Index scores were then divided into 3 groups for the purposes of further analysis. A score of 4 was represented as group ‘a’. A score of 4<score ≤ 8 was represented as group ‘b’. A score of >8 was represented as group ‘c’. To examine the relationship of the 3 groups to survival, a Kaplan-Meier analysis was calculated and shown in Figure 1.

One-year percent survival probabilities were 90.3%, 75.8% and 38.1% for groups a, b and c, respectively. Three-years percent survival probabilities were 51.3%, 43.0% and 10.7% for groups a, b and c respectively. These differences were significant, p<0.0001.

A Cox proportional hazard model for death on the Liver Index score categories (Table 2), showed significant differences in the Hazard Ratios (HR), being 1 for group a (arbitrary reference value), 1.4 for group b (p<0.001) and 4.19 for group c (p<0.001).

### Relationship of liver index and its parameters to HCC aggressiveness

A linear regression model was constructed of the Aggressiveness Index score on the Liver Index score (Table 3A). We found a positive association between the Aggressiveness Index and the Liver Index. For each unit of change in the Liver Index, there was a quarter of a score increase in the Aggressiveness Index (0, 25). A multiple regression model was also calculated for all the 4 parameters of the Liver Index (GGTP, total bilirubin, albumin and platelets) together in the model (Table 3B). We found that 3 of the individual components used for the construction of the Liver Index are positively associated with the Aggressiveness Index. In contrast, however, only the Albumin showed an inverse association, that is, with an increase in albumin levels, the Aggressiveness Index decreased. For all of these components there was a statistically significant association (for all, p<0.001).

We then constructed a table to show the relationship between the Liver Index groups a, b and c and the Aggressiveness Index scores (Table 4). Considering the relationship between the two indices, Aggressiveness Index and Liver function Index, shown in Table 4, we found that there was a positive and significant linear trend between them. The average index values of the Aggressiveness Index increased with each increase in the Liver index categories and the relative differences between these average values were all significant (p<0.001, test for trend), with the exception of the first two categories (‘a’ vs. ‘b’) where the differences were very small. Thus, there was a statistically significant association between the Aggressiveness index, as an expression of tumor aggressiveness, and the Liver index, as an indicator of liver function.’

**Figure 2:**
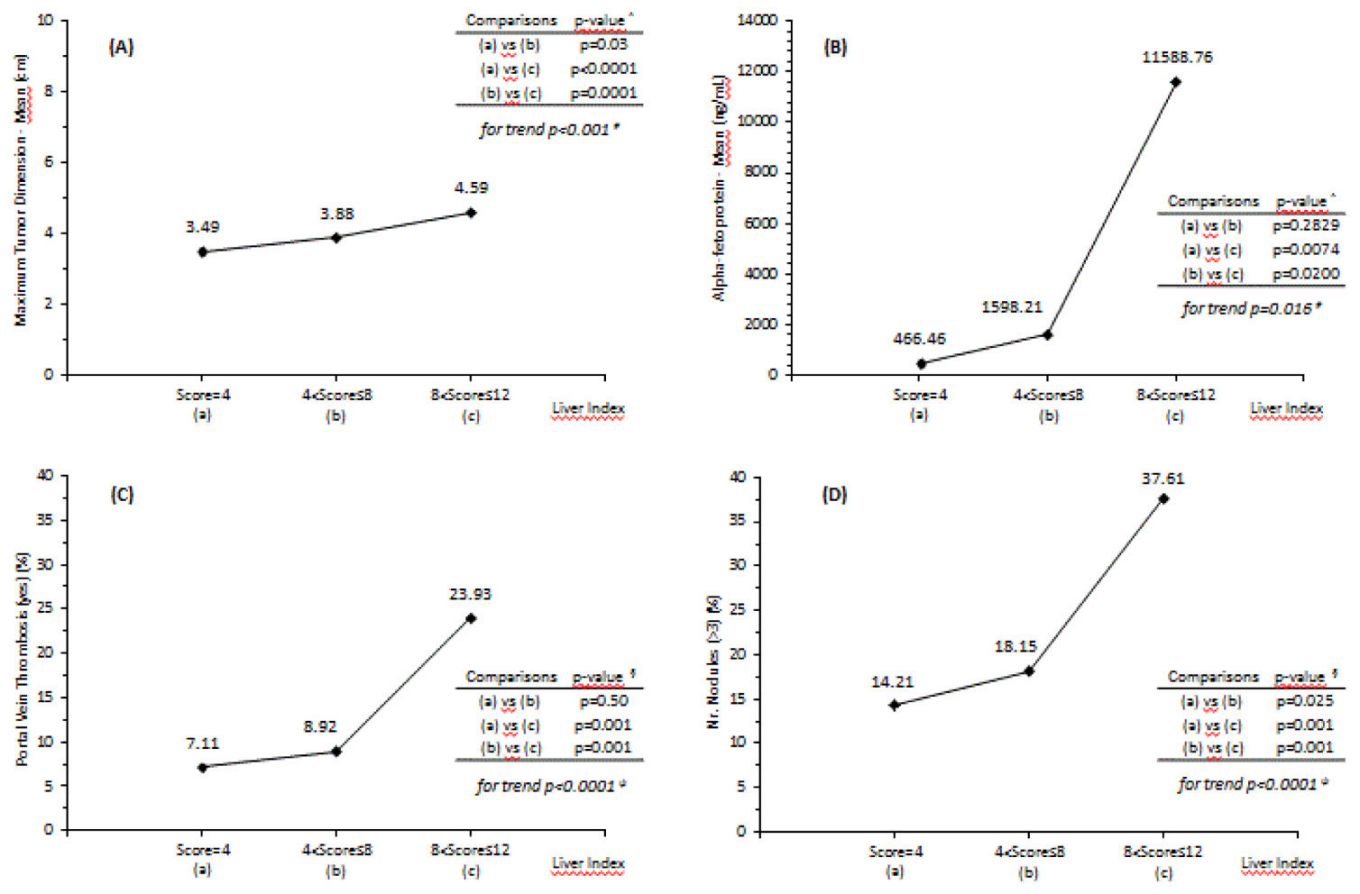
A) Trends between Liver Index score in categories and: Maximum Tumor Dimension (cm); B) Alpha-fetoprotein (ng/mL); C) Portal Vein Thrombosis (yes) (%); D) Number Nodules (>3) (%). #Test z for trend; ^Mann-Whitney test; ψChi-square test for trend; §Multiple comparisons of proportions.

Furthermore, we examined the trends in the relationships between the Liver Scores and the individual components of the Aggressiveness Index (Figure 2) and found that for each of MTD, PVT, multifocality and AFP, there was a significant trend between the Liver Index score and the tumor parameter measures.

## Discussion

It has been long accepted that the prognosis of HCC patients depends on both tumor extent or biology, as well as on liver disease severity [3] and these twin sets of influences have been incorporated into most modern staging systems [4–6]. The liver disease component has recently been modified by inclusion of the semi-quantitative ALBI (albumin and bilirubin) score [7], as well as the independent role in prognosis that systemic inflammation has been shown to have for many tumor types, including HCC, as measured by the serum levels of C-reactive protein and albumin in the Glasgow score [8]. Serum albumin levels thus seem to reflect both systemic inflammation (and nutritional deficiency), as well as synthetic liver function and may even have a role in protection against HCC growth [9].

The 4 parameters that were incorporated into our Liver Index, namely, albumin, total bilirubin, platelets and GGTP, were those that had the highest Hazard Ratios when we examined an HCC Aggressiveness Index in relation to liver function parameters [2]. This large HCC cohort has been extensively characterized [1,2,10]. The Liver Index is comprised of the sum of the scores for these 4 parameters (Table 1) and relates to patient survival, as shown in the Kaplan-Meier and Cox analysis (Figure 1 and Table 2). Elevated levels of serum bilirubin are a predominant marker of liver damage or failure. In the context of HCC, elevated bilirubin levels may result either from HCC invasion and replacement of liver parenchyma, or of liver parenchymal damage from the underlying hepatitis or cirrhosis [10]. Platelet levels, in addition to their role in hemostasis, have been shown to be a marker of or surrogate for cirrhosis [11,12]. It is in this context that they can be viewed in our index. However, they have been recently been considered, as part of the HCC microenvironment, to be involved in HCC growth and in HCC drug resistance [13–17]. Furthermore, platelet levels have recently been considered to be a reflection of 2 HCC phenotypes, based on cirrhosis (small HCCs) or its absence (larger HCCs) [18,19]. GGTP levels, in addition to their use in monitoring hepatic damage in disease, also reflect HCC prognosis; there may also be HCC-specific isoforms and they may be a useful marker in low alpha-fetoprotein HCC phenotypes [20–23].

The Aggressiveness Index was then evaluated with respect to the Liver Index and its 4 components, in a Linear Regression Model and a Multiple Linear Regression model, respectively (Table 3). The Aggressiveness Index was positively associated with the Liver Index, as well as with its components, except for albumin, that was negatively associated, suggesting a protective effect for albumin. We further considered the relationship between the Aggressiveness and Liver Indices (Table 4) and found a positive and significant linear trend between them. We were concerned that at high bilirubin levels, the Liver Index might simply reflect liver destruction by growing tumor mass. We therefore re-calculated the Liver Index using a maximum bilirubin of 3.0 mg/dl. The results were almost identical in the relationship of the Liver Index to the Aggressiveness Index. We then examined the trends in the 3 Liver Index scores in relationship to the Aggressiveness Index components and found a positive and significant trend, when comparing the lowest to the highest Liver Index scores.

It is of interest to consider the possible mechanisms underlying the relationships between liver function tests and indices of HCC biology. Possibly, the changes in liver parameters and the Liver Index with increased tumor aggressiveness simply reflect liver damage as a consequence of parenchymal liver invasion and destruction by HCC. However, given the recent demonstrations of an influence of the underlying liver on HCC biology and prognosis [24,25], there may be effects of the liver on the HCC cells, either non-specifically (and the Liver Index parameters are merely reflections of that) or by direct interactions between bilirubin, albumin, GGTP and platelets (or cirrhosis, for which they are a surrogate marker) and HCC cells. Albumin has already been shown to be protective against HCC growth [9], while platelets can produce HCC mitogens and inflammatory cytokines [13,17,26]. GGTP has been recognized as a marker of experimental hepatocarcinogenesis [27] and may also be involved in HCC growth and drug resistance [28,29]. Doubtless other liver function parameters may also be involved in HCC growth or aggressiveness biology, including microenvironmental inflammatory cytokines and immune cells. Bilirubin has been thought of as an index of residual liver function in HCC patients [30] and to relate to alpha-fetoprotein levels [31].

In conclusion, liver function parameters and the Liver Index correlated with HCC Aggressiveness and may be directly involved in the biology (growth and invasiveness) of HCC cells. However, this Index will need to be externally validated.

## Figures and Tables

**Table 1:** Liver Index as sum of the respective scores.

	Score		
	1	2	3
GGTP (IU/ml)	<100	100 – 200	>200
Total Bilirubin (mg/dl)	<1.5	1.5 – 2.5	>2.5
Albumin (g/dl)	>3.5	2.5 – 3.5	<2.5
Platelets (×10^9^/l)	<100	100 – 150	>150

The Liver Index is the sum of the scores for GGTP+Bil+Alb+Platelets: GGTP (cut-off): GGTP<100; 100 ≤ GGTP ≤ 200; GGTP>200; scores 1, 2, 3 respectively. Bil (cut-off): Bil<1.5; 1.5 ≤ Bil ≤ 2.5; Bil>2.5; scores 1, 2, 3 respectively. Alb (cut-off): Alb>3.5; 2.5 ≤ Alb ≤ 3.5; Alb<2.5; scores 1, 2, 3 respectively. Platelets (cut-off): Plt<100; 100 ≤ Plt ≤ 150; Plt>150; scores 1, 2, 3 respectively The Liver Index score was divided into three groups for Kaplan-Meier Survival plots and Cox analysis: a, score=4; b, 4<score ≤ 8; and c, score>8.

GGTP- Gamma glutamyl transpeptidae; Bil- total bilirubin; Alb- Albumin; Plt- Platelets.

**Table 2:** Cox proportional hazard model in HCC patients for death on Liver Index Score categories in total cohort.

		HR*	se(HR)	p	95% C.I.	Comparison s of HRs	p
Liver Index							
Score=4 [Ref. category]	(a)	1	--	--	--	(a) vs (b)	<0.001
4<Score ≤ 8	(b)	1.41	0.13	<0.001	1.17 to 1.70	(a) vs (c)	<0.001
8<Score ≤ 12	(c)	4.19	0.54	<0.001	3.26 to 5.40	(b) vs (c)	<0.001

* Hazard Ratio;

Liver Index groups: a, score=4; b, 4<score ≤ 8; and c, score>8.

Liver Index is the sum of the scores for GGTP +Bilirubin + Albumin +Platelet GGTP (cut-off): GGTP<100; 100 ≤ GGTP ≤ 200; GGTP>200; for scores 1, 2, 3 respectively.

Bilirubin (cut-off): Bil<1.5; 1.5 ≤ Bil ≤ 2.5; Bil>2.5; for scores 1, 2, 3 respectively.

Albumin (cut-off): Alb>3.5; 2.5 ≤ Alb ≤ 3.5; Alb<2.5; for scores 1, 2, 3 respectively.

Platelet (cut-off): Plt<100; 100 ≤ Plt ≤ 150; Plt>150; for scores 1, 2, 3 respectively

**Table 3:** A) Linear regression model of Aggressiveness Index score on Liver Index score, B) multiple linear regression model on all parameters Platelets, GGTP, total Bilirubin and Albumin together in the model.

	β	Se(β)	p	95% C.I.
(A)				
Liver Index score	0.2462	0.0247	<0.001	0.1978 to 0.2945
(B)				
GGTP (IU/ml)	0.0013	0.0003	<0.001	0.0007 to 0.0020
Total Bilirubin (mg/dl)	0.0585	0.0140	<0.001	0.0311 to 0.0859
Albumin (g/dl)	−0.3821	0.0554	<0.001	−0.4908 to −0.2733
Platelets (x10^9^/L)	0.0031	0.0005	<0.001	0.0021 to 0.0041

Aggressiveness Index (sum of scores): MTD (in tertiles): MTD<4.5; 4.5 ≤ MTD ≤ 9.6; MTD>9.6; for scores 1, 2, 3 respectively; AFP (cut-off): AFP<100; 100 ≤ AFP ≤ 1000; AFP>1000; for scores 1, 2, 3 respectively; PVT (No/Yes): PVT(No); PVT(Yes); for scores 1, 3 respectively; Nodules (number): Nodules ≤ 3; Nodules>3; for scores 1, 3 respectively.

Liver Index is the sum of scores: GGTP (cut-off): GGTP<100; 100 ≤ GGTP ≤ 200; GGTP>200; for scores 1, 2, 3 respectively. Bilirubin (cut-off): Bil<1.5; 1.5 ≤ Bil ≤ 2.5; Bil>2.5; for scores 1, 2, 3 respectively. Albumin (cut-off): Alb>3.5; 2.5 ≤ Alb ≤ 3.5; Alb<2.5; for scores 1, 2, 3 respectively. Platelets (cut-off): Plt<100; 100≤Plt ≤ 150; Plt>150; for scores 1, 2, 3 respectively.

MTD- Maximum Tumor Diameter; AFP- Alpha-fetoprotein; PVT- Portal Vein Thrombosis; GGTP- Gamma glutamyl transpeptidae; Bil- Total bilirubin; Alb- Albumin; Plt- Platelets.

**Table 4:** Relationship between Liver Index groups a, b and c and Aggressiveness Index Score, in the total cohort.

Liver Index Score								
		Score=4	4<Score≤8	8<Score≤12	pF	Comparisons	p°	p¥
		(a)	(b)	(c)				
	N	197	1636	117				
Aggressiveness Index (M ±SD)	4.92 ± 1.15	5.19 ± 1.51	6.26 ± 1.91	0.0001	(a) vs (b)	0.11	<0.001	
						(a) vs (c)	<0.0001	
						(b) vs (c)	<0.0001	

F Kruskal-Wallis rank test;

° Wilcoxon rank-sum (Mann-Whitney) test;

¥ Test for trend. Abbreviations as in Table 3.

Aggressiveness Index (sum of scores): MTD(cm) (in tertiles): MTD<4.5; 4.5 ≤ MTD ≤ 9.6; MTD>9.6; for scores 1, 2, 3 respectively; AFP (ng/ml) (cut-off): AFP<100; 100 ≤ AFP ≤ 1000; AFP>1000; for scores 1, 2, 3 respectively; PVT (No/Yes): PVT(No); PVT(Yes); for scores 1, 3 respectively; Nodules (number): Nodules≤3; Nodules>3; for scores 1, 3 respectively.

The Liver Index (sum of scores): GGTP (IU/ml) (cut-off): GGTP<100; 100 ≤ GGTP ≤ 200; GGTP>200; for scores 1, 2, 3 respectively. Bilirubin (mg/dl) (cut-off): Bil<1.5; 1.5 ≤ Bil ≤ 2.5; Bil>2.5; for scores 1, 2, 3 respectively. Albumin (g/dl) (cut-off): Alb>3.5; 2.5 ≤ Alb ≤ 3.5; Alb<2.5; for scores 1, 2, 3 respectively. Platelets (×10^9^/L) (cut-off): Plt<100; 100 ≤ Plt ≤ 150; Plt>150; for scores 1, 2, 3 respectively Liver Index score was divided into three groups: group a, score=4; group b, 4<score ≤ 8; and group c, score>8.

## Abbreviations

HCC: Hepatocellular Carcinoma
AFP: Alpha-Fetoprotein
GGTP: Gamma Glutamyl Transpeptidase
PVT: Portal Vein Thrombosis
MTD: Maximum Tumor Diameter
CT: Computerized Axial Tomography Scan
Alb: Albumin
Plts: Platelets
Bil: Bilirubin
